# Anticancer Compound Plumbagin and Its Molecular Targets: A Structural Insight into the Inhibitory Mechanisms Using Computational Approaches

**DOI:** 10.1371/journal.pone.0087309

**Published:** 2014-02-27

**Authors:** Mohammad S. Jamal, Shadma Parveen, Mohd A. Beg, Mohd Suhail, Adeel G. A. Chaudhary, Ghazi A. Damanhouri, Adel M. Abuzenadah, Mohd Rehan

**Affiliations:** 1 King Fahd Medical Research Center, King Abdulaziz University, Jeddah, Kingdom of Saudi Arabia; 2 Bareilly College, M.J.P. Rohilkhand University, Bareilly, U.P., India; 3 KACST Technology Innovation Center in Personalized Medicine, King Abdulaziz University, Jeddah, Kingdom of Saudi Arabia; Wayne State University School of Medicine, United States of America

## Abstract

Plumbagin (5-hydroxy-2-methyl-1,4-naphthoquinone) is a naphthoquinone derivative from the roots of plant *Plumbago zeylanica* and belongs to one of the largest and diverse groups of plant metabolites. The anticancer and antiproliferative activities of plumbagin have been observed in animal models as well as in cell cultures. Plumbagin exerts inhibitory effects on multiple cancer-signaling proteins, however, the binding mode and the molecular interactions have not yet been elucidated for most of these protein targets. The present study is the first attempt to provide structural insights into the binding mode of plumbagin to five cancer signaling proteins viz. PI3Kγ, AKT1/PKBα, Bcl-2, NF-κB, and Stat3 using molecular docking and (un)binding simulation analysis. We validated plumbagin docking to these targets with previously known important residues. The study also identified and characterized various novel interacting residues of these targets which mediate the binding of plumbagin. Moreover, the exact modes of inhibition when multiple mode of inhibition existed was also shown. Results indicated that the engaging of these important interacting residues in plumbagin binding leads to inhibition of these cancer-signaling proteins which are key players in the pathogenesis of cancer and thereby ceases the progression of the disease.

## Introduction

**Figure 1:**
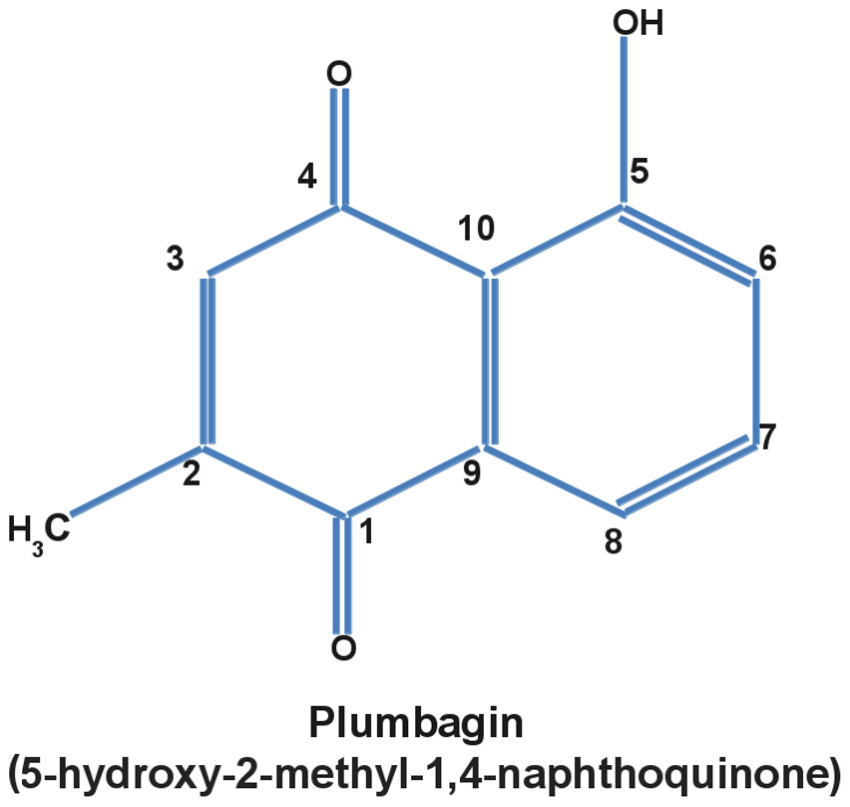
Two dimensional structure of plumbagin. The IUPAC name of the plumbagin is '5-hydroxy-2-methyl-1,4-naphthoquinone'. The carbon atoms are shown with the IUPAC numbering. The numbers are referred in the text wherever a particular atom of plumbagin is involved in molecular interaction with target proteins.

Cancer is the leading cause of disease worldwide accounting for 12.7 million new cases every year and this number is expected to rise to a whopping 26 million by 2030 [1]. Considering the impact on human health and economics, cancer presents a major challenge to the scientific world and there is a necessity to discover novel agents for the treatment of this disease. Several studies have been focused on naturally occurring chemical compounds which are known to possess cytotoxic effects and have the potential for killing cancer cells [2]–[3]. Plumbagin (PL; 5-hydroxy-2-methyl-1,4-naphthoquinone, Figure 1) is one such important compound, which is a naphthoquinone derivative identified from the roots of plant *Plumbago zeylanica* and belongs to one of the largest and diverse groups of plant metabolites [4]–[6]. The anticancer and antiproliferative activities of PL have been observed in animal models as well as in cell cultures [7]–[12]. The anticancer activities of PL have been shown against a wide variety of cancers including breast cancer [13], lung cancer [14]–[15], ovarian cancer [16], acute promyelocytic leukemia [17], and prostate cancer [18]–[19]. The inhibitory effect of PL was shown by alterations of various signaling pathways which play a crucial role in cancer cell proliferation, survival, invasion, and metastasis [14], [16], [18], [20]–[22] through suppression of major signaling molecules such as nuclear factor-kappaB (NF-κB) [23], AKT/mTOR [13], and signal transducer and activator of transcription 3 (Stat3) [24].

In the current study, we considered five key signaling molecules namely phosphatidyl inositol-4,5-bisphosphate 3-kinase (PI3K), AKT (also known as PKB, protein kinase B), anti-apoptotic protein B cell lymphoma-2 (Bcl-2), NF-κB, and Stat3 which play important role in cancer development and progression. These molecules have already been reported [13], [18], [23], [25] to show direct inhibition by PL. Owing to their key role in cancer development, these regulatory molecules are frequently targeted in anticancer therapy.

The PI3K and AKT pathway, commonly known as PI3K/AKT/mTOR pathway, is perhaps the most frequently dysregulated pathway in human cancers [26]–[29]. Consequently, significant efforts have been made to generate inhibitors of the key kinases in PI3K pathway including PI3K, AKT, and mTOR in recent years [30]. Plumbagin targets PI3K in human breast cancer cells and drastically decreases the level of PI3K subunit p85 causing downstream AKT/mTOR pathway inhibition leading to growth arrest and cell death [13], [25]. The PL target, AKT, plays an important role as antiapoptotic protein [31], [32] and is a widely exploited for anticancer therapy [33]–[35]. Plumbagin inhibited AKT kinase activity and induced autophagy in two cancer cell lines, MDA-MB-231 and MCF-7 [13].

The signaling molecule NF-κB transcription factor plays a major role in the development and progression of various types of cancer [36]–[38]. A constitutive and continuous NF-κB activity is observed in various cancer tumors including lymphoid or myeloid tumors [39]–[42]. In addition, tumor cells often exploit NF-κB to achieve resistance to chemo- and radio-therapy [43]. Anti-cancer therapies have been developed by exploiting the inhibitors of NF-κB which may reduce the progression and development of the disease and/or may improve the efficacy of conventional therapies [44]. In this regard, certain inhibitors were discovered which suppress activity of NF-κB by directly blocking its binding to DNA [45]–[47]. Using the similar mechanism, PL inhibits NF-κB activity by interfering with the binding of DNA to NF-κB as demonstrated by gel shift assay of nuclear proteins [23], [25].

The Bcl-2 family of proteins are the principal regulators of programmed cell death or apoptosis [48], [49]. The over-expression of Bcl-2 has been observed in various cancers which contributes to drug resistance [50] and enhances in-vivo cell survival [51] while reduction in Bcl-2 expression increases drug sensitivity [52]. Several reports [53]–[58] have explored the development of inhibitors of Bcl-2 protein as potential anti-cancer drugs. In this regard, plumbagin has also been shown to induce apoptosis by down-regulation and inactivation of Bcl-2 in human breast cancer cells [25].

The signaling molecule Stat3 is constitutively activated in many human cancers and has been widely exploited as therapeutic target for cancer therapy [59]–[63]. In this regards, plumbagin has been shown [18] to inhibit the DNA-binding activity of Stat3 in prostate cancer cell lines, DU145, PC-3, and CWR22rv1.

Although PL mediated inhibition of the aforementioned five signaling molecules has been reported as reviewed above, the binding mechanisms and the molecular interactions of PL with these key cancer regulatory molecules is apparently not known. Plumbagin docking has been reported previously with few protein molecules [16], [64]–[68], however, the present study is the first attempt to investigate the structural and molecular details of PL binding against five key cancer signaling targets viz. PI3Kγ, AKT1/PKBα, Bcl-2, NF-κB, and Stat3 using molecular docking and ligand (un)binding simulation approach. In addition, we also identified and ranked some important interacting residues of these target proteins.

## Materials and Methods

### Data retrieval

The molecular structure of PL was retrieved from PubChem compound database with CID 10205. The 3-D structures of five chosen cancer signaling targets were retrieved from Protein Data Bank (PDB) and are: AKT1 (PDB ID: 3O96), Bcl-2(PDB ID: 2O21), NF-κB (PDB ID: 3GUT), PI3Kγ (PDB ID: 3L54), and Stat3 (PDB ID: 1BG1). The bound ligand was used as probe for the binding site grid generation. In case of Stat3 protein, the available structure is from mouse and differs from the human homolog in an amino acid residue at 760 position (Asp instead of Glu in human). The Stat3 structure contains 3D atomic coordinates until the amino acid residue at 716 position. Therefore, being 100% identical within the whole range of amino acids for which 3D coordinates are available, it will presumably serve as Stat3 structure for human as well. All the software tools used in this study are summarized in Table 1.

**Table 1:** The computational tools which were used in this study are presented with their source and application.

Computational tool	Availability	Application
Dock v.6.5	http://dock.compbio.ucsf.edu/	Molecular docking of ligand into the binding site of the receptor.
PyMOL v.1.3	http://www.pymol.org/	Molecular visualization and analysis
LigPlot+	http://www.ebi.ac.uk/thornton-srv/software/LigPlus/	Molecular interaction between receptor and ligand.
Naccess v.2.1.1	http://www.bioinf.manchester.ac.uk/naccess/	Loss in accessible surface area of residues
X-Score v.1.2.11	http://sw16.im.med.umich.edu/software/xtool/	Binding energy and dissociation constant of ligand
MoMA-LigPath	http://moma.laas.fr	Simulate protein-ligand (un)binding

### Molecular docking

Dock v.6.5 from the University of California, San Francisco was used for all computer simulations and docking of PL into the active site pockets of five chosen cancer drug targets was achieved [69]. The best conformation search strategy exploited in the present work was random conformation search, which utilized the existing Coulombic and Lennard-Jones grid-based scoring function. The whole process of docking involves multiple steps. Briefly, once the potential site of interest is identified on the receptor, a grid is generated within the site and each grid point is considered as a sphere center. The orientation of the ligand within the site of interest is calculated using a set of matching atom-sphere pairs. To evaluate the orientation of the ligand within the site, a shape scoring function and/or a function approximating the ligand-protein binding energy is used. The ligand-protein binding energy is an approximate sum of the van der Waals attractive, van der Waals dispersive, and Coulombic electrostatic energies. As a final step, to minimize the energy score, the orientation of the ligand is varied slightly and evaluated. After the initial orientation and evaluation (scoring) of the ligand, a simplex minimization is used to reach the nearest local minimum of the energy score. The orientation of the ligand corresponding to the nearest local minimum energy score serves as the final docked ligand.

### Analysis of docked protein-ligand complex

PyMOL v.1.3 [70] was used to analyze and generate an illustration of whole protein-ligand complex. LigPlot+ v.1.4.3 program [71]–[72] was used for analyzing the interaction of docked protein-ligand complex to check the polar and hydrophobic interactions between the receptor and ligand and illustrations of molecular interactions between PL and the chosen proteins were generated. To confirm the involvement of interacting residues obtained from LigPlot+, loss in Accessible Surface Area (ASA) was calculated. If a residue lost more than 10 Å^2^ ASA in the direction from the unbound to the bound state, it was considered to be involved in interaction [73]. The ASA of unbound protein and the protein-ligand complex were calculated using Naccess v.2.1.1 [74]. The change in ASA (ΔASA) of the *i* ^th^ residue in the direction from unbound to bound state was calculated using the expression:



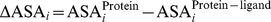



In addition to the Dock score (Grid score) obtained from Dock [69], the binding energies and dissociation constants were also calculated by X-Score v.1.2.11 [75]–[76].

### Protein-ligand (un)binding simulation

MoMA-LigPath (http://moma.laas.fr), a web server which works on Molecular Motion Algorithms (MoMA) [77]–[78], simulates the ligand unbinding from the binding site of the protein to the surface of the protein. It considers the flexibility for the protein side-chains and the ligand, and involves only geometric constraints. This method provides mechanistic information about how ligand is driven to the binding site from the surface of the protein or from the binding site to the surface. It also provides snapshots of molecular interactions leading the ligand from surface of the protein to the binding site. In the process, it identifies those residues which despite being away from the binding site still play important role in ligand binding or in driving the ligand to the binding site. The docked molecular complex was subjected to (un)binding simulation using MoMA-LigPath.

## Results and Discussion

### Plumbagin docking and (un)binding simulation study of PI3Kγ

**Figure 2:**
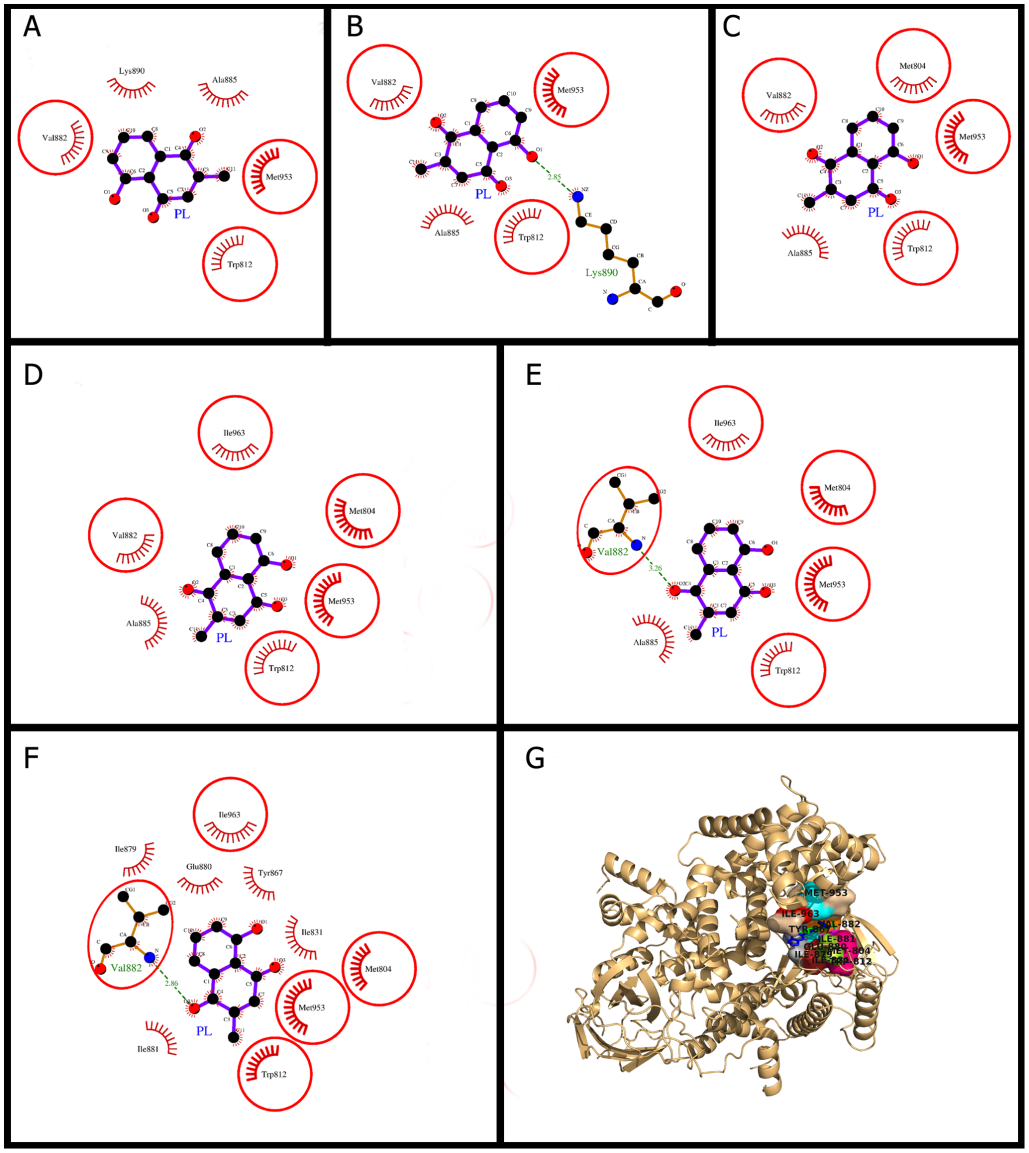
Plumbagin (PL) binding to the cavity of PI3Kγ. Panels A–F: show the (un)binding simulation phases of PL, 'A' is farthest from the binding site, 'E' is the closest to the binding site, and 'F' is the binding site phase. The hydrogen bonds are shown as green-dashed lines with indicated bond length and the residues involved in hydrophobic interactions are shown as red arcs. Those residues which are common to the last phase (F) are encircled. Panel G: Another representation for phase F. The whole protein is displayed in cartoon representation and the ligand molecules are in sticks; PL colored as green and the bound known inhibitor in blue. The interacting residues are labeled and shown as surface in different colors.

The snapshots of molecular interactions leading the ligand from surface of the protein to the active site are shown in Figure 2A–G. Final binding in the active site and the molecular interactions holding PL in the active site are shown (Figure 2F-G, Table 2). Plumbagin packs against the residues Ile-963, Met-953, Val-882, Ile-881, Glu-880, Ile-879, Tyr-867, Ile-831, Trp-812 and Met-804 (Table 3). In the final docking phase of PI3Kγ, a hydrogen bond was formed between O-atom of 1-carbonyl group of PL and α-amino group of Val-882 and other 34 hydrophobic interactions were from 10 different residues (Figure 2F, Table 2, Table 3). The Dock score was highly negative and binding energy was also comparable to that of the known inhibitor while the dissociation constant was approximately one tenth of the known inhibitor (Table 2). The residues Trp-812, Val-882, and Met-953 were common in all the phases of PL (un)binding simulation, showing their importance in initial recruitment of PL from surface of the protein to the deep binding site (Figure 2). Previously, the residues of PI3Kα subunit viz. Ile-800, Ile-848, Val-850, and Val-851, (the equivalent residues in PI3Kγ are Ile-831, Ile-879, Ile-881 and Val-882 respectively) were also reported to play a role in binding of the inhibitor wortmannin. Specifically, the residue Val-851 (equivalent to Val-882 of PI3Kγ) was involved in hydrogen bonding to wortmannin through its amide group [79]. Also in an earlier report [80], a highly potent inhibitor GSK2126458 of PI3Kγ was co-crystallized with the protein (PDB IDs: 3L54, 3L08) in which Val-882 was involved in hydrogen bonding. In another study [81], Val-882 through its backbone was also found to be involved in hydrogen bonding with H-bond acceptor in five different inhibitors i.e. wortmannin, LY294002, quercetin, myricetin, and staurosporine. The wortmannin was found to be packing itself against N-terminal lobe residues of PI3Kγ viz. Ile-831, Ile-879, Ile-881, and Val-882 [81] which are overlapping with the PL binding site. The hydrogen-bonding feature by the residue Val-882 was reported to be conserved in all kinase-inhibitor complexes [82]. In this regard, our docking simulations of PL and PI3Kγ were in agreement with the above studies e.g., the conserved H-bonding feature of Val-882 and the other common residues involved in PL binding. These consistent findings provided validation for our molecular docking and also showed that PL inhibits PI3Kγ with the mechanism similar to those of other reported inhibitors.

**Table 2:** The binding strength of plumbagin (PL) with the five cancer signaling proteins is shown with number of molecular interactions and other scores.

Target	PDB ID	Hydrogen bonds	Hydrophobic interactions	Dock/Grid score	Binding energy for PL (kcal/Mol)	-log(K_d_) for PL	Binding energy for known inhibitor (Kcal/Mol)	-log(K_d_) for known inhibitor
PI3Kγ (p110)	3L54	1	34 (10)	−26.85	−7.73	5.66	−9.57	7.02
AKT1	3O96	0	27 (6)	−28.40	−7.62	5.58	−11.69	8.57
Bcl-2	2O21	0	22 (5)	−19.86	−6.66	4.88	−10.82	7.93
NF-κB (p65)	3GUT	2	11 (4)	−19.53	−6.84	5.02	-	-
Stat3	1BG1	1	11 (6)	−20.14	−6.39	4.69	-	-

The number of residues involved in the hydrophobic interactions are provided in parentheses. The 'K_d_' denotes the dissociation constant. The binding energy and −log(K_d_) values are calculated using X-Score. The more negative is the Dock/Grid score, the better is the docking.

**Table 3:** The plumbagin interacting residues of the five cancer signaling proteins are listed with the number of non-bonding contacts and the loss in Accessible Surface Area (ASA).

Target	Plumbagin interacting residues	No. of Non-bonded contacts	ΔASA (in Å^2^)
PI3Kγ (p110)	Met-804	1	15.69^5^
	Trp-812*	2	20.37^4^
	Ile-831	3	24.31^2^
	Tyr-867	8	6.19^9^
	Ile-879	1	12.18^7^
	Glu-880	2	4.92^10^
	Ile-881	2	7.61^8^
	Val-882 (H-bonding)*	4	12.45^6^
	Met-953*	7	31.39^1^
	Ile-963	4	22.66^3^
AKT1	Trp-80*	20	59.53^1^
	Ser-205	1	16.08^5^
	Leu-210	1	19.41^2^
	Thr-211	2	12.79^6^
	Tyr-272	1	18.42^3^
	Asp-292	2	18.36^4^
Bcl-2	Thr-93	1	17.78^3^
	Gln-96	1	18.31^2^
	Ala-97	2	14.31^5^
	Tyr-199*	17	47.08^1^
	Pro-201	1	16.46^4^
NF-κB (p65)	Tyr-36*	4	26.13^2^
	Val-121	1	4.21^5^
	Lys-122	2	7.40^4^
	Lys-123 (H-bonding)*	4	36.19^1^
	Asn-155 (H-bonding)	0	17.53^3^
Stat3	Arg-382	3	17.63^5^
	Glu-415	1	17.80^2^
	Arg-417 (H-bonding)*	2	42.93^1^
	Cys-418*	3	17.74^3^
	Gly-419*	1	6.91^6^

The residues involved in hydrogen-bond formation are indicated by 'H-bonding' in parentheses. The residues were also ranked on the basis of loss in solvent accessibility indicated by superscripts with the value of ΔASA.

*The residues which were common across all phases of (un)binding simulation.

### Plumbagin docking and (un)binding simulation study of AKT1

**Figure 3:**
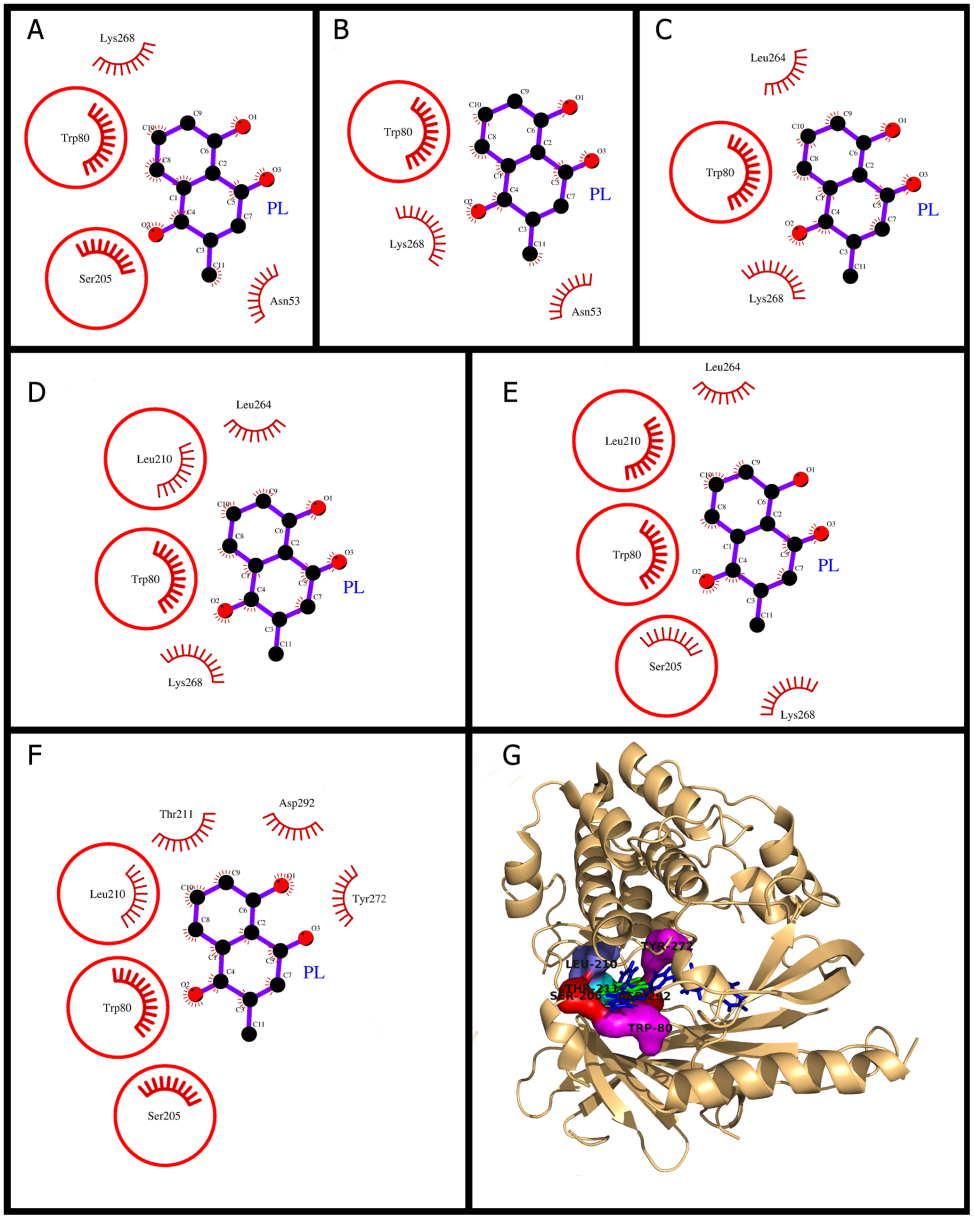
Plumbagin (PL) binding to the cavity of AKT1. Panels A–F: show the (un)binding simulation phases of PL, 'A' is farthest from the binding site, 'E' is the closest to the binding site, and 'F' is the binding site phase. The residues involved in hydrophobic interactions are shown as red arcs. Those residues which are common to the last phase (F) are encircled. Panel G: Another representation for phase F. The whole protein is displayed in cartoon representation and the ligand molecules are in sticks; PL colored as green and the bound known inhibitor in blue. The interacting residues are labeled and shown as surface in different colors.

The mechanistic study of PL binding provided information of varying molecular interactions with respect to decreasing distance from binding site (Figure 3A–E). The analysis of docking of AKT1 has revealed that PL binds deep inside the cavity and is stabilized by the hydrophobic interactions (Figure 3F–G). However, the binding energy and dissociation constant of PL was lesser than the bound inhibitor (Table 2, Table 3). This may occur due to the smaller size of PL than the bound inhibitor. The Dock score was also highly negative and number of hydrophobic interactions that kept PL bound in the cavity was also reasonably high (27 interactions from 6 different residues) as mentioned in Table 2. The key residue in AKT1 for PL binding was Trp-80, which was common through all phases of PL (un)binding simulation, demonstrating its importance in initial binding of PL and finally bringing it into the active site (Figure 3). The Trp-80 was also involved in the majority of hydrophobic interactions and showed highest decrease in its solvent accessibility after PL binding (approx 60 Å^2^) as shown in Table 3. This finding was consistent with a study [83] in which it was shown that the inhibition by Akti (an inhibitor of AKT) is critically dependent upon a solvent-exposed tryptophan residue (Trp-80) present in all three AKT isoforms and whose mutation to an alanine yields an Akti-resistant kinase. It was observed that the Trp-80 acts as the key interacting residue in docking and (un)binding simulation study of PL suggesting that PL also inhibits AKT1 by the mechanism similar to that of Akti inhibitor and, therefore, validated our docking predictions for AKT1 as well. The Akti acts as non-ATP-competitive allosteric inhibitor and blocks AKT phosphorylation and does not lead to active phosphorylated state of AKT [83] and thus the PL also inhibits AKT1 by the similar mechanism i.e., acts as non-ATP-competitive allosteric inhibitor. The inhibitory mechanism of PL (similar to Akti) is in contrast to ATP-competitive inhibitors, which protect the phosphorylated site of AKT from dephosphoryation by phosphatases [83].

### Plumbagin docking and (un)binding simulation study of Bcl-2

**Figure 4:**
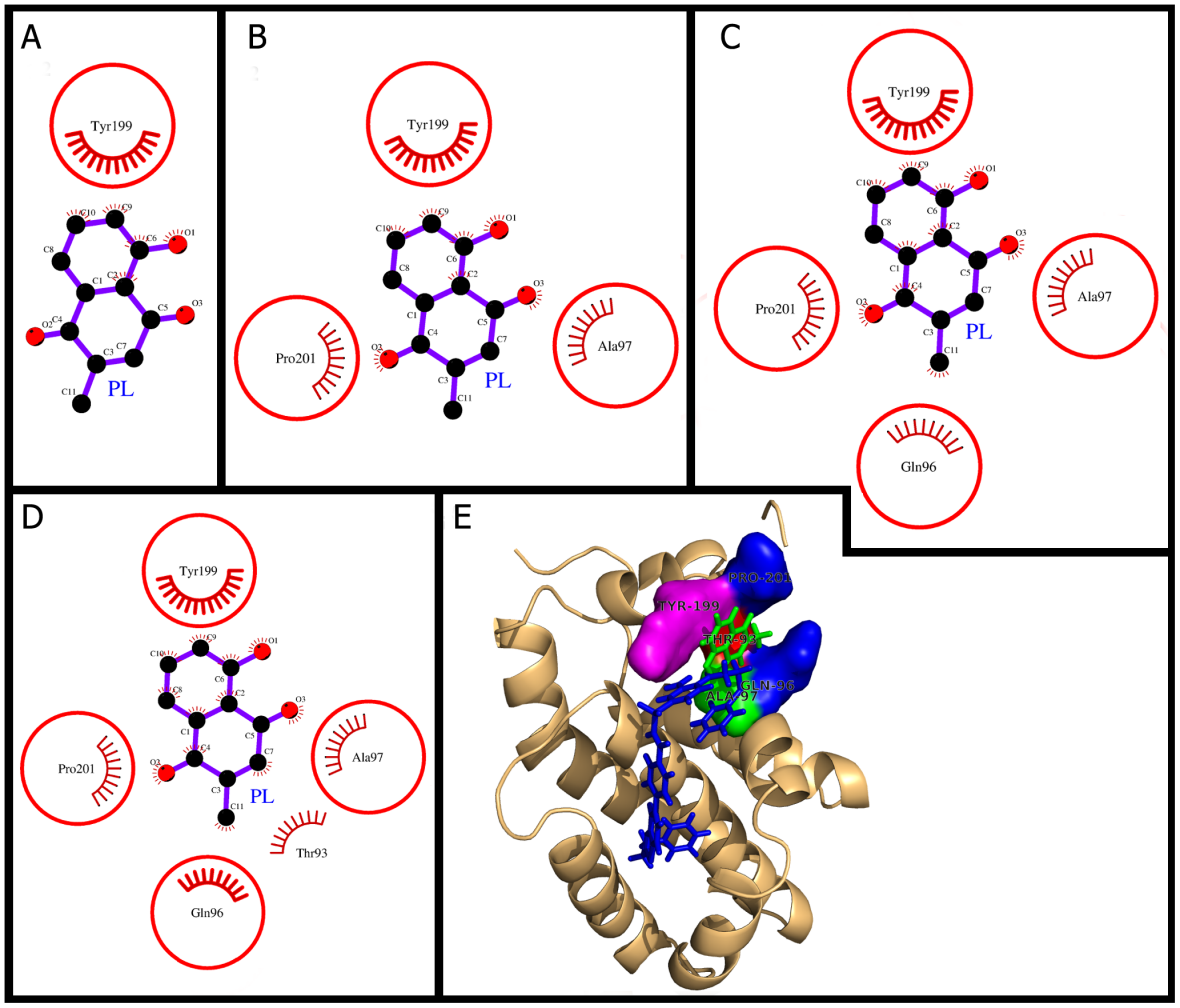
Plumbagin (PL) binding to the cavity of Bcl-2. Panels A–D: show the (un)binding simulation phases of PL, 'A' is farthest from the binding site, 'C' is the closest to the binding site, and 'D' is the binding site phase. The residues involved in hydrophobic interactions are shown as red arcs. Those residues which are common to the last phase (D) are encircled. Panel E: Another representation for phase D. The whole protein is displayed in cartoon representation and the ligand molecules are in sticks; PL colored as green and the bound known inhibitor in blue. The interacting residues are labeled and shown as surface in different colors.

The (un)binding simulation phases of PL with increasing number of molecular interactions while approaching towards the binding site are shown in Figure 4A–D. The docking phase (Figure 4D-E) showed molecular interactions of Bcl-2 with PL which binds on the exterior region of Bcl-2 cavity using hydrophobic interactions. Owing to the large size of previously known Bcl-2 inhibitor (acyl-sulfonamide-based ligand) [84] in the complex structure (PDB ID: 2O21), the binding constant and binding energy of PL was much smaller than that of the inhibitor (Table 2). In previous studies of molecular docking of inhibitor compounds, the residue Tyr-199 of Bcl-2 was involved in hydrogen bonding [85], [86]. However, in the present study, Tyr-199 was not involved in hydrogen bonding, rather this residue was involved in maximum number of hydrophobic interactions and lost maximum surface accessibility (47 Å^2^) while switching to bound state (Figure 4D, Table 3). The Tyr-199 was also found to be common in all phases of (un)binding simulation of PL which again shows its importance for PL binding (Figure 4). All the observations for Tyr-199, such as the maximum number of hydrophobic interactions involved, the maximum loss in surface accessibility, and the common residues in (un)binding simulation were corroborating with one another. Furthermore, the key residue involved, Tyr-199, was also same as observed in the previous docking studies with other inhibitors [85]-[86].

### Plumbagin docking and (un)binding simulation study of NF-κB

**Figure 5:**
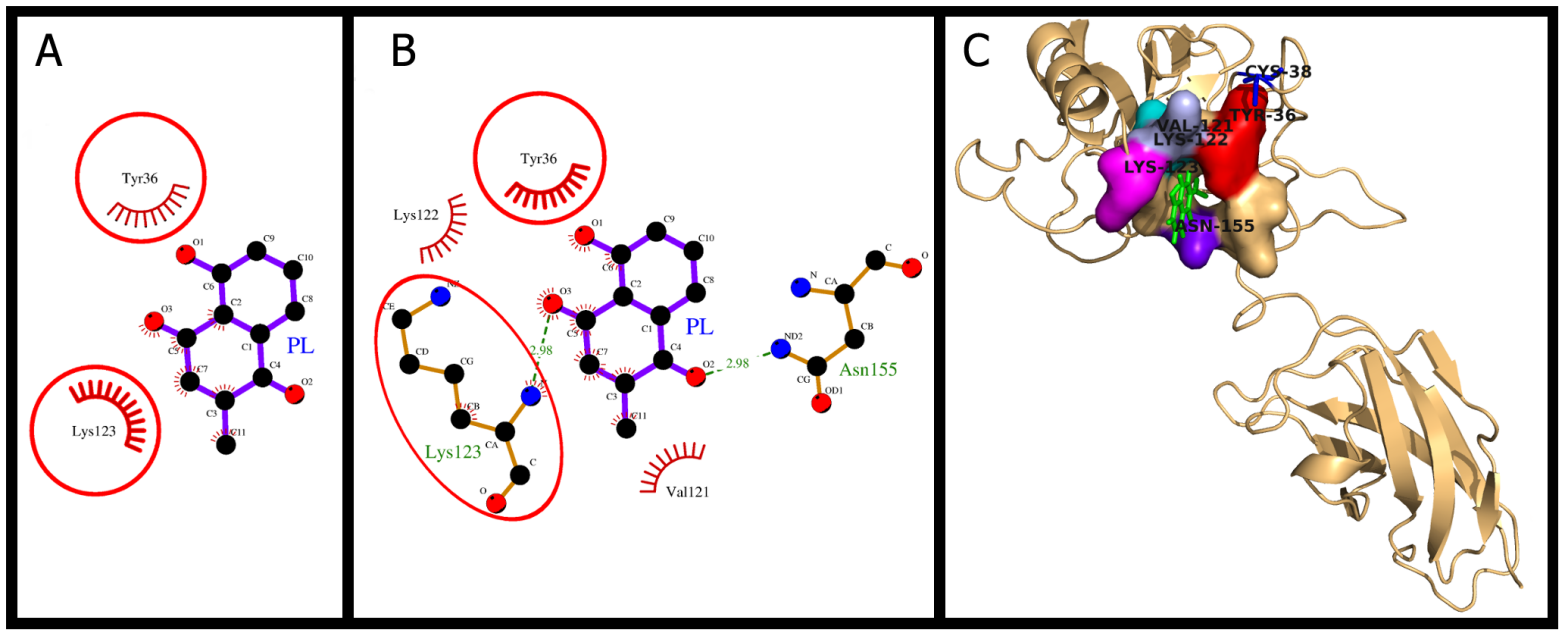
Plumbagin (PL) binding to the cavity of NF-κB. Panels A-B: show the (un)binding simulation phases of PL, 'A' is farthest from the binding site and representing many stages always coming with 2 interacting residues and 'B' is the binding site phase. The hydrogen bonds are shown as green-dashed lines with indicated bond length and the residues involved in hydrophobic interactions are shown as red arcs. Those residues which are common to the last phase (B) are encircled. Panel C: Another representation for phase B. The whole protein is displayed in cartoon representation and PL in sticks colored as green. The interacting residues are labeled and shown as surface in different colors.

The molecular docking and (un)binding simulation study of NF-κB with the interacting residues and molecular interactions are shown in Figure 5A-B. The first phase (Figure 5A) represents multiple stages of binding where the interacting residues were same but PL is approaching towards binding site and at variable distance. In the molecular docking phase (Figure 5B-C), PL packed against the residues Lys-123, Lys-122, Val-121, Tyr-36, and Asn-155. As shown in the Figure 5B, PL formed two hydrogen bonds, one between O-atom of 1-carbonyl group of PL and N-atom of amide group of Asn-155 (2.98 Å) and the other between O-atom of 4-carbonyl group of PL and α-amino of Lys-122 (2.98 Å). There were 11 hydrophobic interactions from 4 different residues (Table 2, Table 3). It has been reported [87] that the Lys-122 and Lys-123 of NF-κB p65 subunit are the only acetyl-acceptor lysines which contact with DNA in minor groove as revealed from the crystal structure of the p50/p65 heterodimer bound to DNA. The acetylation of these residues reduces κB-DNA binding (may be neutralized by the positive charge on Lys-122 and Lys-123 ε-amino groups) and represses its transcriptional activity, and thus leads to attenuation of p65-mediated transcription [87]. In our study, of the two lysine residues, Lys-122 formed H-bond and Lys-123 was involved in non-bonding contact with PL. Lys-123 was also found common among all phases of (un)binding simulation study (Figure 5). Therefore, the lysine residues (Lys-122, Lys-123) were not available for DNA binding, which resulted in the reduction in the DNA binding activity. The Dock score, the binding energy, and the dissociation constant were also comparable with the docking results of other chosen targets (Table 2).

### Plumbagin docking and (un)binding simulation study of Stat3

**Figure 6:**
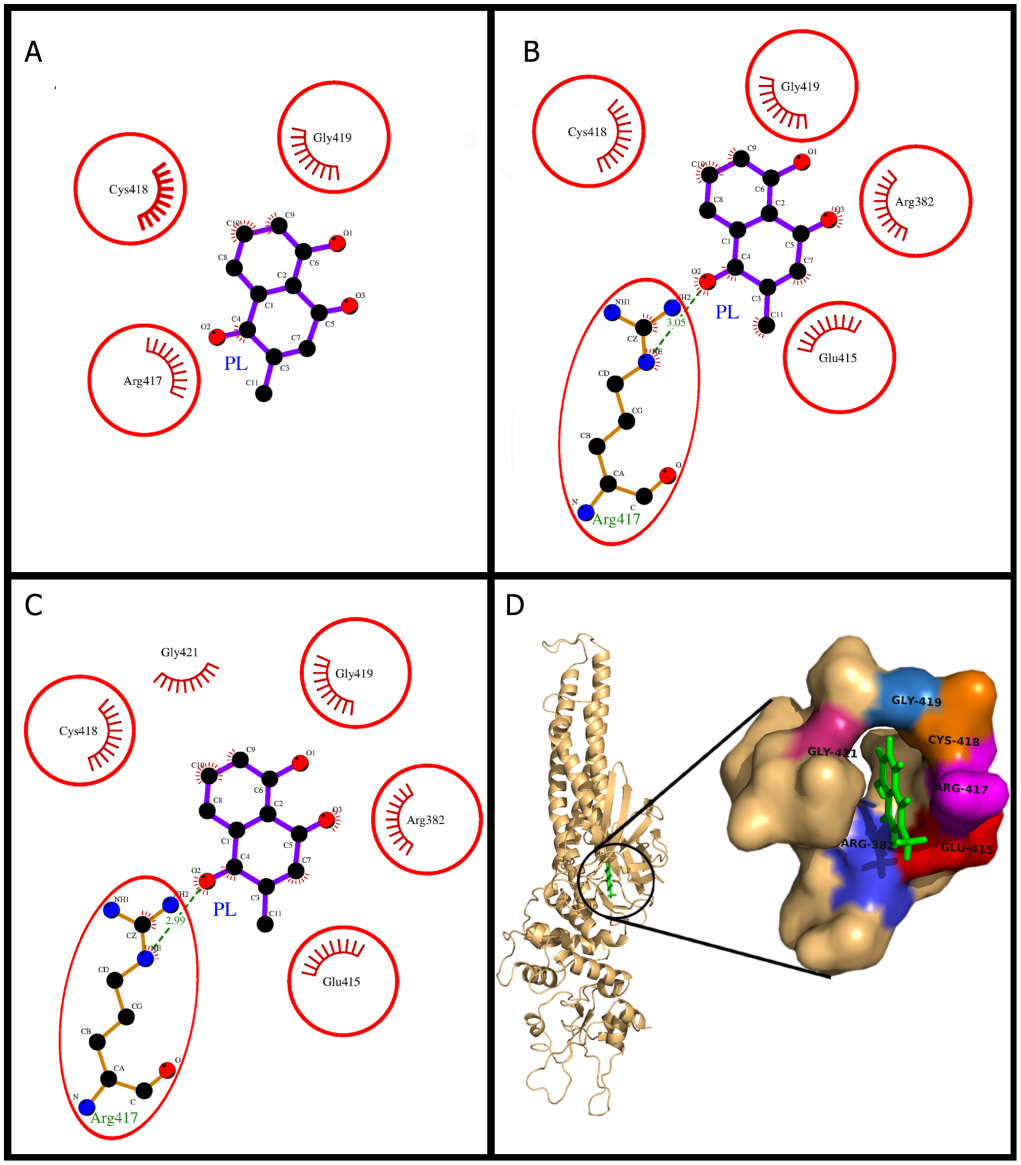
Plumbagin (PL) binding to the cavity of Stat3. Panels A–C: show the (un)binding simulation phases of PL, 'A' is farthest from the binding site, 'B' is the closest to the binding site, and 'C' is the binding site phase. The hydrogen bonds are shown as green-dashed lines with indicated bond length and the residues involved in hydrophobic interactions are shown as red arcs. Those residues which are common to the last phase (C) are encircled. Panel D: Another representation for phase C. The whole protein is displayed in cartoon representation and PL in sticks colored as green. The interacting residues are labeled and shown as surface in different colors.

The (un)binding simulation phases with the increasing molecular interactions are shown in Figure 6A–C. The docking phase (Figure 6C-D) showed that PL bound to the DNA binding domain and packed against the residues Gly-421, Gly-419, Cys-418, Arg-417, Glu-415, and Arg-382. The Arg-417 was involved in hydrogen bonding interaction through the N_ε_ atom of its guanidium group (Figure 6C) and also showed maximum loss of solvent accessibility in PL bound state (Table 3). Other residues involving hydrophobic contacts also showed significant loss in solvent accessibility in switching from unbound to bound state (Table 3). A mutational analysis study [88] created the dual mutation Arg-414/417 that led to complete loss of DNA binding activity of Stat3. The NH1 of guanidium group of Arg-417 is involved in hydrogen bonding with phospho-diester bonds of bases T-1007 and T-2007 (from complementary strand) [89]–[90]. Similarly, another residue Arg-382, is also reported to be involved in hydrogen bonding with phospho-diester bonds of bases T-1006 and T-2006 (from complementary strand) [89]–[90]. In another study, it has been reported that the residue Arg-382 when mutated to Trp and Gln abrogated the DNA-binding ability of Stat3 homo-dimer [91]. In our study, Arg-417 with 2 other residues (Cys-418, Gly-419) was found to be common throughout all phases of (un)binding simulation study (Figure 6). The residues Arg-417 (H-bonding, maximum loss of solvent accessibility, common in (un)binding simulation) and Arg-382 (hydrophobic contacts) play important role in PL binding, which is in agreement with the aforementioned studies. The Dock score is also highly negative comparable to the docking results of other chosen targets. The binding energy and the dissociation constant for PL were also similar to the results of other chosen targets (Table 2).

## Conclusions

The present study for the first time used docking and (un)binding simulation analysis to identify interacting residues of five important cancer-signaling proteins and their molecular interactions with PL. Plumbagin inhibited the molecules by involving their functionally important residues leading to the loss of function. Among various important residues identified during the docking analysis, the key residues for the chosen proteins that play major role in PL mediated inhibition are: the PI3Kγ residue, Val-882, involved in the hydrogen-bonding interaction; the AKT1 residue, Trp-80, showing highest decrease in the solvent accessibility after PL binding; the Bcl-2 residue, Tyr-199, showing maximum hydrophobic interactions; the NF-κB residue, Lys-122, involved in H-bond formation; and the Stat3 residue, Arg-417, forming H-bond. The exact mode of inhibition was indicated when multiple modes of inhibition existed (i.e. AKT1). In all the five signaling target molecules, the Dock score was highly negative, which showed good quality docking. We also presented the exact binding of PL and identified and characterized various interacting residues within the active sites of respective chosen proteins. In addition, we ranked the importance of residues for PL binding using ASA analysis. Also, the importance of residues was shown by ASA analysis (higher loss in solvent accessibility) and interaction analysis (high number of hydrophobic interactions or presence of H-bond) and by their consistent appearance in (un)binding simulation phases. A peculiar observation in this study was that PL formed hydrogen bond interactions with the target proteins only through the oxygen-atom of 1-carbonyl group which acted as a strong hydrogen donor. This finding may be useful for increasing drug potency in lead optimization where PL will serve as starting lead compound. The current study of PL docking analysis provides the structural insights into the binding mechanism of PL to the five chosen cancer signaling molecules. The PL-protein architecture is expected to guide scientists as a suitable model for understanding the multi-targeting drug structure and in providing structural details for the inhibitory mechanisms.
